# The X-Ray in Dentistry

**Published:** 1911-09-15

**Authors:** J. M. King

**Affiliations:** Nashville, Tenn.


### THE X-RAY IN DENTISTRY.

BY J. M. KING, M.D., NASHVILLE, TENN.

Read before the Tennessee State Dental Association, May 24th, 1911.

I do not feel that it is at all necessary to go into the details of the X-ray before this association as to its generation, apparatus, etc., believing that it is sufficient to only call attention to the use of the X-ray in diagnosis and to explain the technique of making radiographs.

One of the most practical uses of the X-ray is in dentistry. The greater density of the teeth enables them to be shown well in contrast with the bone of the jaw, and with certain quality of ray the tooth itself is transparent and its pulp-cavity and root-canals may be studied, and even pulp-stones may be discovered.

A fluoroscopic examination will frequently reveal all that is to be known concerning a case, but it is preferable as a rule to make a radiograph, which is done in short time—ordinarily within ten or fifteen minutes. Celluloid films cut the desired size and wrapped in black paper and sealed may be held in the mouth behind the teeth or abscess cavity to be studied and the X-ray turned on from a suitable tube for three or four seconds. The exposed piece of film is then developed by the same method for developing and fixing kodak films. By this means a permanent record is made of the existing condition.

The examination of the films may be made by transmitted light by holding them before an open window. In normal tissue the soft parts will appear dark, the bone lighter, the teeth still lighter with the canal a little darker than the body of the tooth. The regular illuminating box in the radiographic room may be used, also.

The conditions in dentistry that may be advantageously studied by the aid of the X-ray being rather numerous,

I think it is best to take up each one separately and give the respective diagnostic points.

*Unerupted Teeth*: It is frequently necessary to ascertain whether or not there are unerupted teeth. This is so easily and satisfactorily done by the technique above described.

The unerupted superior canine is usually found to be present and generally lies more nearly parallel with the median line than normally. If it presses against the root of the lateral incisor a cyst cavity is often to be seen there. The unerupted third molar is almost always found and frequently impacted.

The other molars and the bicuspids, if much delayed in eruption, are fully as apt to be found entirely absent as to be present. Tousey states that it is a serious mistake to extract a persistent temporary tooth in any of these positions before using the X-ray.

A question sometimes arises as to whether a certain tooth is a persistent temporary molar or a permanent bicuspid. The X-ray will determine and if it is a persistent temporary tooth it will show whether or not the germ of the permanent tooth is present or absent.

X-ray examinations have shown that absorption of the roots of temporary teeth takes place without the pressure of permanent teeth.

Tousey cites a remarkable case which was unearthed by the X-rays. The patient, twenty years of age, was going about with three sets of teeth in his mouth. He had retained all of his temporary teeth but they gave him such an infantile expression that he had made a set of artificial teeth. An X-ray examination revealed a full set of unerupted permanent teeth.

*A cyst* or *abscess* cavity in the bone shows as a dark area on the film, with clearly defined outline.

*Necrosis* shows a darker area on the film, but the interpretation must be made by one familiar with radiography of this kind.

*Necrosis* and *fistula* may be shown by the X-ray to be due to a broken root or to a fragment of root-filling. The fillings show white in a film.

*Fracture of the inferior maxilla* may require study, especially an ununited fracture.

*Root fillings* are often the subject of investigation by the X-ray. Imperfection may be shown by the opacity not extending to the apical foramen, or by breaks occurring along the length of the filling, or by the filling extending beyond the apical foramen or through the lateral wall of the tooth.

The location of the cause of a *chronic fistula* may be revealed if two or three regions are suspected.

The extent of *Rigg's disease* may be observed by a radiograph.

The X-ray is of service in locating the *root-canal* and *apical foramen*, in cases of commencing alveolar abscess where the dentist's drill fails to find the proper route to the cavity.

The radiograph will show whether or not the *pulp of the canal* has been completely removed to the extremity of the root, it will also show the *fracture* of a root, and also the *curvature* of the root.

The buccal roots are the most difficult to show, unless they have root filling.

It is sometimes necessary to ascertain whether the roots penetrate the *antrum* and this may be done with the X-ray.

Sometimes the roots of the temporary teeth fail to be absorbed and thus cause a deflection of the oncoming permanent set. The X-ray would show the unabsorbed root.

The demand for the X-ray is growing daily in its therapeutic as well as in its diagnostic use in general surgery, and when its great value becomes familiar and well recognized by the dentists and dental surgeons, there will be a still greater demand for it. The information obtained by the X-ray is so positive that the course to pursue in a given case may be determined at once without any hesitation or delay.

### DISCUSSION.

Dr. Gordon White: One of Dr. King's illustrations was that of a lady in a very nervous condition and very much concerned about the result of the operation for the removal of the third molar. Her husband and her mother were both interested in it and were in my office at the time of the operation. When the lady made her appearance we found an imbedded right superior third molar had turned somewhat, the cusp had enlarged right under the neck of the second molar, exposing both the gum and side, with the grinding face more or less against the surface of the second molar. As the lady was in an exceedingly nervous condition, it seemed to me very necessary that I know every detail of what I was doing when I undertook to remove the tooth. Dr. King gave me the radiograph the next day, and to my very great pleasure I could see the outline of the surrounding neighborhood, not every feature, but the exact position of the teeth.

I want to say that nothing has ever given me in my whole practice as much satisfaction as that one operation. After seeing the radiograph I knew that it could be very easily removed with an ordinary elevator. That elevator was made of a broken hatchet shaped excavator, which had been sharpened for that purpose twenty years ago, and which I had used very successfully. This lady, as I said, was exceedingly anxious about herself, her husband also was exceedingly anxious about her, and it became very necessary, as I remarked, to know what I was doing. She brought with her her own pyhsician, the doctor who was to attend her in her confinement, and I suggested to him that before he made any injection whatever hyperdermically that he give her a little morphine, he said that was a good idea, and he gave her a dose of morphine, and in a few moments later the injection of the anesthetic. I took my homemade elevator and flipped that third molar out of there in a twinkle, and the result was I made an everlasting friend and a new patient.

It is almost impossible to tell the limit of the good we can receive by the use of the X-ray. I recently had a man with a lower wisdom tooth that had been giving him a great deal of trouble. I did not know what he had in there, but he suffered excruciatingly. I sent him also to Dr. King to have the X-ray made of this lower third molar. To my very great surprise I found by looking at the X-ray that there was practically no inflammation there, no broach or filling or anything else through the roots as I expected. He was the most extreme neurotic that I think I have ever seen in my practice and it was with great difficulty that I persuaded him to allow the tooth to remain, though what I did do was simply to try to give him relief. I told him that he was exceedingly susceptible to pain, but that he must be patient, that I would fill that root after a few days' treatment and we could save the tooth, if he would only be patient and suffer for a little bit. I treated the tooth, relieved it, and filled the root, and he hasn't had any trouble since.

I do not know how much of this work I have had done, I have had others besides Dr. King, but I want to say, however, that none has been so satisfactory as his, and it has been worth a great deal in my practice. The X-ray is going to be much more generally used in every field of surgery, and for dentistry it is more especially useful, because I think we are working in the most obscure and delicate field of surgery. I believe that it is a most valuable adjunct that we are going to get better acquainted with by the necessities demanding it. I think that we are going to be able to do better scientific work, and certainly better technical work. We shall have knowledge of what the diagnosis may be to say the least. I think the field for X-ray work, as I said awhile ago, is almost unlimited.

Dr. Meadows: It is more to thank Dr. King, for being with us than for any other reason that I am on the floor. I will not occupy much of your time except to tell of one recent experience that I had regarding an incisor which was delayed in eruption. The mother was very much troubled. Governed by the schooling which I had received from our old friend, Dr. Crawford, whenever you find delayed permanent teeth, remove always the temporary teeth, so I informed the mother that that permanent tooth was there, but for some reason the temporary incisor had not been shed, it was best for the child to have the first removed and allow the permanent tooth to come down in its proper place. In short, I directed her to have an X-ray made of the case, and a few days afterwards she brought it in just delighted, and said, "Doctor, that tooth is there, I see it, and it was not long before the tooth was being erupted of its own accord. The only thing that was needed was the removal of that temporary tooth.

Dr. Rhea: I wish to thank the Doctor for his contribution, and I am sure that we all appreciate it, and have learned something from it. There seems to be an impression among the laity that the X-ray is dangerous. They seem to think that awful results accompany the exposure to X-rays. I do not know just how general this opinion is, however, I have met with it in one case especially where I tried to send a lady to have an X-ray made of her face. She was willing, but her husband was bitterly opposed to it and he wouldn't stand for it at all, and I could not persuade him. I would like for Dr. King to say something to-night in regard to the danger of this X-ray exposure, which I do not believe exists.

Dr. L. G Noel: I have been very much interested for the last four or five years in the theurapeutic value of the X-ray in the treatment of diseased conditions of the teeth, and also the treatment of the epithelium. Dr. King has successfully treated two cases in my family, my grandmother and a friend of mine, and they were indeed successful, and I would like to have Dr. King tell us whether the X-ray has any therapeutic value in the treatment of any diseased condition of the teeth, or peridental membrane. There is one use we might make of it, and that is this; no matter how skillful we are, some times we get hold of a bad broach and break it off in the root and we work to try to get it out, but fail, because we do not know where it is, and how deep it is, and it might possibly be some service to us in realizing it, if we could make a good radiograph of it and take it home and study its position and how it is situated. We do not know the position of it and if we had a good radiograph we might work more intelligently, to say the least of it.

Dr. T. M. Hudson: For a thorough diagnosis it is very helpful. I know in my own instance where I have seen it used in locating the position of the teeth that have been delayed in their eruption. In addition to that I want to ask Dr. King if the X-ray has been beneficial in locating an old sinus where you can't get it located by a probe. The reason I ask, I know a case now where a lady has been in my office several times who has a case where the sinus has made an opening in the external surface of the lower jaw, and we can use a probe for a little distance and still cannot come to the seat of the trouble. I want to ask Dr. King if his X-ray can locate a thing of that kind.

Dr. Henry W. Morgan: Mr. President, I want to bear my testimony to the efficiency of radiographs. I have had some very satisfactory results from it. I had a patient a few months ago, who was sent to me by a neighboring dentist who had been working with it for about three years trying to heal it, a sinus in the mouth of a child about twelve years of age. I took the patient to Dr. King and he located by the radiograph an abscessed upper right lateral and to the right bicuspid. The case was opened, the pulp devitalized, the pus discharged, and the communication between the pulp chamber and the sinus cleansed with a salt solution, and the patient referred back to the home dentist for treatment. The efficiency of the X-ray in locating delayed teeth, especially those of the cuspid variety, occasionally where the child has lost a temporary central and after several years there is no appearance of the approach of the permanent central, it is always wise to ascertain exactly the relationship of the permanent teeth to the others, and in more than one case we have been enabled by paring away the alveolar process to bring out these delayed teeth. I want to go on record as being opposed to the extraction of deciduous teeth without first having a radiograph of them. I have seen more than one instance in which the teeth were crowded together by the too early extraction of the deciduous teeth, and it became necessary afterwards to apply orthodontic measures to get the teeth in their proper positions which would have otherwise come down normally if there had not been too much haste in extracting the deciduous teeth.

Dr. King: Mr. Chairman, first in reference to the danger from the X-ray. For the time you expose the patient in taking a radiograph, it is only for a second and there can not possibly be any danger attributed to it. As a matter of course you can produce dangerous burns by the continued exposure to X-rays, but in this exposure for the radiograph you cannot possibly do so. In taking the X-ray picture of a tooth there is absolutely no danger and no pain to the patient. The patient need have no fear at all, you take a film and expose it behind the tooth and it is over in an instant.

So far as the therapeutics question is concerned. I was reading a few days ago where a fistulous tract had healed after exposure to the X-ray. It had been treated with the most careful attention beforehand, and the doctor in writing upon this subject thought that just the exposure to the X-ray had some favorable value. That is the only information that I have on that subject. The fistulous tract healed after this exposure after it had been treated for a long time. But one case would not prove much, I think.

As far as Dr. Hudson's case is concerned in locating the fistulous tract, in all probability it might be located, you might trace it down to a remaining root, or part of a root, and that fistulous tract ought to show up dark in the field of the film and in all probability it could be located.

